# Role of Dedicated Cardiac Emergency Unit in Early Identification and Management of Acute Myocardial Infarction in a Developing Country of South Asia

**DOI:** 10.7759/cureus.11423

**Published:** 2020-11-10

**Authors:** Farhala Baloch, Amina Khan, Ashmal Kabani, Saulat Fatimi, Javed Tai, Aamir H Khan, Shiraz Hashmi, Mazeera Aslam

**Affiliations:** 1 Medicine/Cardiology, The Aga Khan University, Karachi, PAK; 2 School of Nursing, The Aga khan University, Karachi, PAK; 3 Medicine, The Aga Khan University, Karachi, PAK; 4 Cardiothoracic Surgery, The Aga Khan University, Karachi, PAK; 5 Cardiology, The Aga Khan Hospital, Karachi, PAK; 6 School of Nursing, The Aga Khan University, Karachi, PAK

**Keywords:** acute myocardial infarction, cardiac emergency unit, key performance indicators, acute st-elevation myocardial infarction

## Abstract

Background

The care of patients presenting with chest pain to multidisciplinary services hospital gets compromised due to the busy triage system. A separate and specialized equipped cardiac emergency unit (CAR-ERU) can improve patient’s outcomes.

Objectives

To enhance early recognition and treatment of acute myocardial infarction (AMI) patients. To sustain key performance quality indicators (KPIs) for AMI.

Methods

In October 2016, a separate CAR-ERU was established inside the multidisciplinary emergency department (MED). A dedicated specialized heart-lung and vascular teams were hired under the supervision of service line leadership. The KPIs that were identified benchmark with international practice guidelines. Data were collected and stored for analysis. Exemption from the ethical review committee was obtained.

Results

A total of 2914 patients visited CAR-ERU from October 2016 to September 2017 for a period of one year. Out of which 30% were diagnosed with acute coronary syndrome (ACS) and this included 8% diagnosis with ST-segment elevation myocardial infarction (STEMI). Over 98.8% of the electrocardiogram (ECG) was done within 10 minutes of arrival while aspirin was given to 96.5% of patients within one hour. The door to balloon time (DBT) of <90 min was achieved in 70% of patients. A significant reduction in length of stay in the emergency department and financial burden was noted. Sustainability of major KPI was observed over the subsequent years.

Conclusion

The introduction of a dedicated CAR-EU improved clinical outcomes, reduced length of stay and financial burden in AMI patients managed in CAR-EU. Our tertiary care hospital is the first one of its kind to take this quality initiative in a lower-middle-income country (LMIC) Pakistan.

## Introduction

Chest pain accounts for about 5.6 million emergency department (ED) visits annually^ ^[1], Among these, early recognition of ST-segment elevation myocardial infarction (STEMI) cases is critical especially in a multidisciplinary hospital where a common and busy triage system receiving a myriad of patients with different complaints [2,3]. In developing countries, less focus has been given to emergency medical care [4]. The healthcare system in our country is at a progressive stage. Participation in various programs like making health policies, public-private partnerships and many others do exist but with a limited scope and there is a need to take strong initiatives [5].

Multiple evidences suggest that deaths are prevented and disabilities averted for diseases such as acute myocardial infarction (AMI), stroke, pregnancy and sepsis by strengthening emergency services, through the diagnostic protocols, decision aids, novel approach and other system changes [6,7]. AMI is one of the leading causes of death and accounts for 10.6% of hospital mortality and disability worldwide [8,9]. On the other hand mortality in STEMI is clearly related to the time of symptom onset to reperfusion time. Every patient who presents with symptoms suggestive of STEMI must have an electrocardiogram (ECG) done within 10 minutes of first medical contact (FMC) followed by a sequence of intervention including compliance to time-bound aspirin and reperfusion algorithms [10]. Our hospital has a precise vision of providing quality patient care. Although our multidisciplinary emergency department demonstrates encouraging results through organized approaches in terms of provision of prompt quality services, however, patients presenting with chest pain needs a quick response out of a busy triage counter dealing with hundreds of patients and variable symptoms on presentation.

The innovation followed by the implementation of the heart-lung and vascular service line in our institute brought forward a notion to establish a separate dedicated cardiac emergency unit (CAR-ERU) within the existing emergency department which would aim to early identification and treatment of STEMI patients and enhance the quality of care. This quality initiative step of providing a separate CAR-ERU in a multidisciplinary tertiary care hospital to improve patient outcomes is taken the first time in Pakistan.

## Materials and methods

A dedicated cardiac team of cardiac physicians and nurses were allocated for separate CAR-ERU. All patients age 18 and above of either gender presenting with signs and symptoms suggestive of acute myocardial infarction were immediately moved to this unit. The key performance quality indicators (KPIs) were identified that set a benchmark in accordance with international practice guidelines. The data for the KPIs were collected and analyzed by dedicated quality improvement committee (QIC) nurses and research officer. Monthly QIC meetings were conducted under the leadership for feedback and future directions.

The KPIs includes ECG within 10 minutes of FMC which is the emergency department in our scenario, aspirin within 60 minutes of arrival, the proportion of STEMI patients receiving revascularization within 90 minutes, the outcome matrices includes mortality and morbidity, length of emergency stay and cost-effectiveness which ultimately leads to patient’s satisfaction. The initial data for the period of one year from 2016 to 2017 were analyzed on a quarterly basis. The subsequent data to measure the sustainability of major KPIs are analyzed over the year 2018 and compared with 2019 data. 

For continuous variables, we report means and standard deviations for normally distributed variables whereas for non-normally distributed variables we determined frequencies and proportions. Chi-square or Fisher exact test was applied for categorical variables as appropriate. Data were analyzed using Stata version 12 (StataCorp LLC, College Station, USA). A p-value of < 0.05 was deemed significant.

## Results

**Figure 1:**
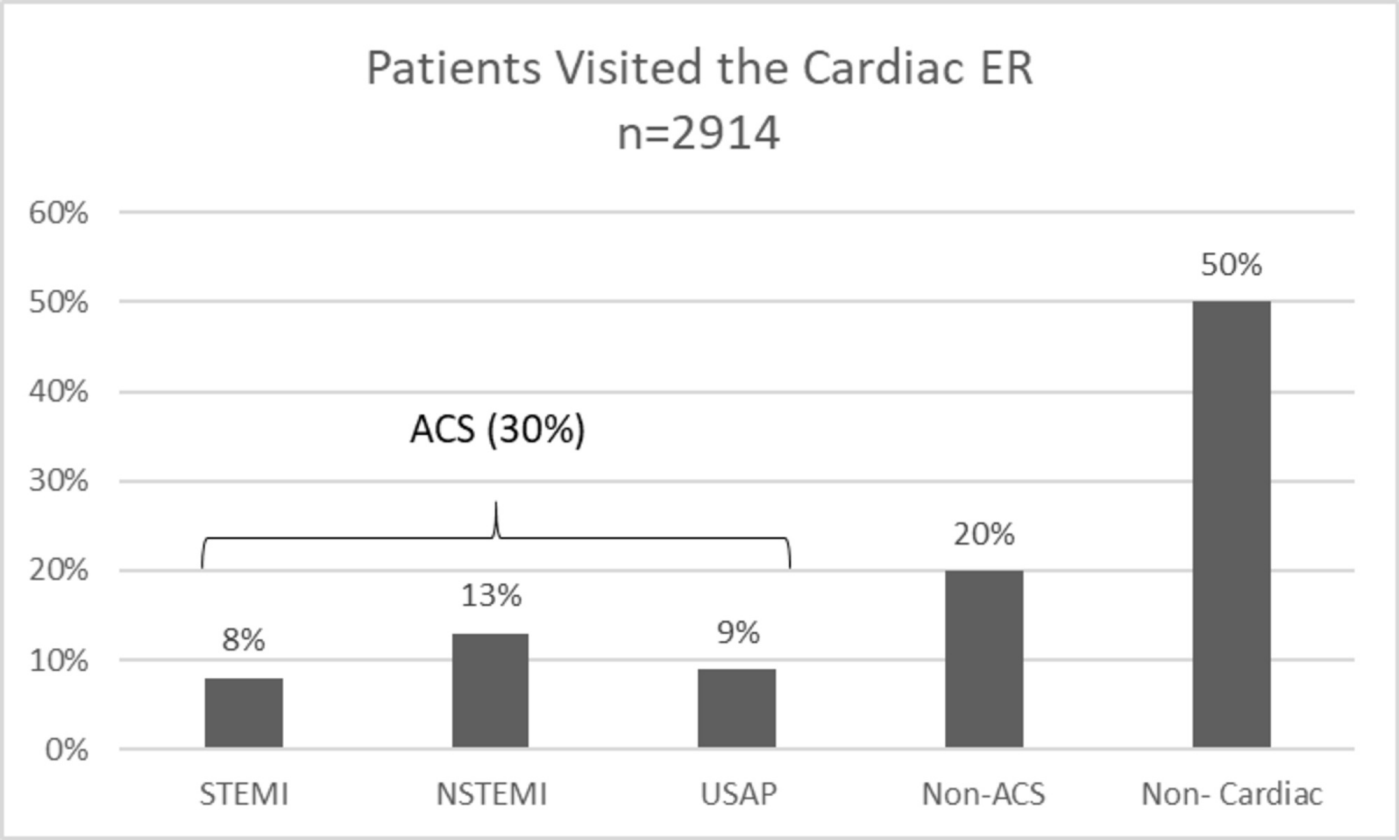
Diagnostic trend of patients admitted in the CAR-ERU CAR-ERU: cardiac emergency unit.

A total of 2914 patients visited CAR-ERU from October 2016 to September 2017. Out of which 30% were diagnosed with the acute coronary syndrome (ACS) and this included 8% diagnosed with STEMI, 13% diagnosed with NSTEMI. Twenty percent were recorded as non-ACS (such as patients with heart failure, valvular disease, arrhythmias and hypertension urgency) cardiac cases (Figure 1).

We found that 50% (n=1457) patients initially suspected of cardiac pathology were finally diagnosed as a non-cardiac case by a specialized and dedicated cardiac team within one hour of their arrival. This resulted in a significant reduction in emergency length of stay of chest pain patients and subsequently the cost paid by the patient. In STEMI, on an average 98.8% of ECGs were done within 10 minutes of arrival while the first dose of aspirin was given within 60 minutes to an average of 96.5% of the patients.

**Figure 2:**
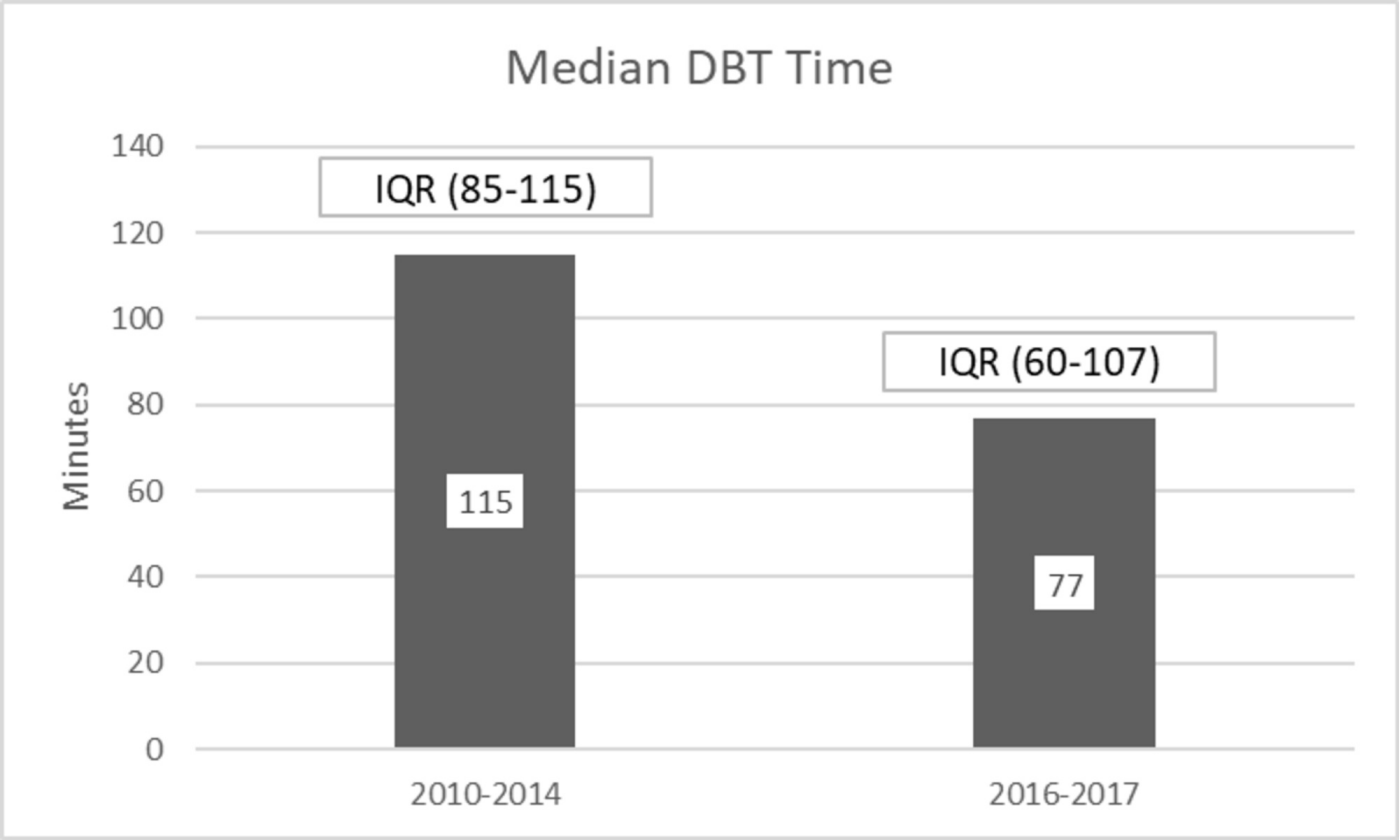
Comparison of median DBT time of STEMI patients DBT: door to balloon time, STEMI: ST-segment elevation myocardial infarction.

The door to balloon time (DBT) for the STEMI patients was less than 90 minutes in 70% of patients; the international benchmark is 90% according to the national cardiovascular data registry. The median DBT of CAR-ERU patients was 77 minutes (IQR 60-107 minutes) as compared to our previous years (2010-2014) institutional audit data before the establishment of CAR-ERU showing median DBT of 115 minutes IQR 85 - 155 with less than 50% of patients had DBT less than 90 min as shown in Figure 2.

**Figure 3:**
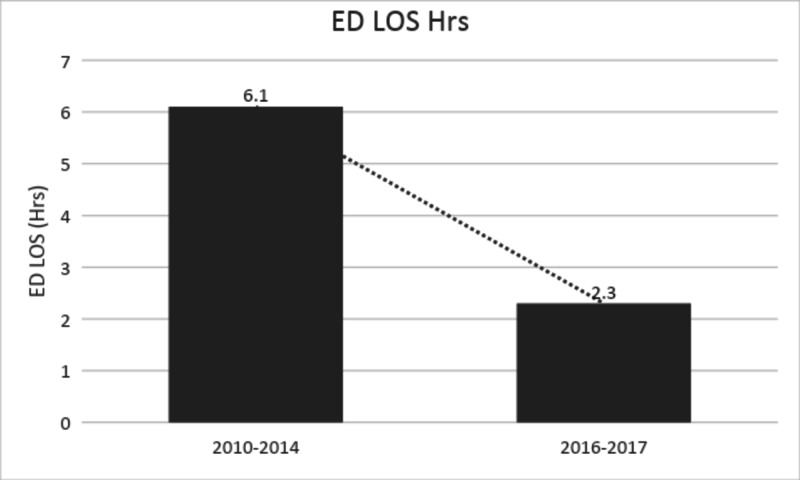
Trend in ED length of stay by year range ED: emergency department.

**Figure 4:**
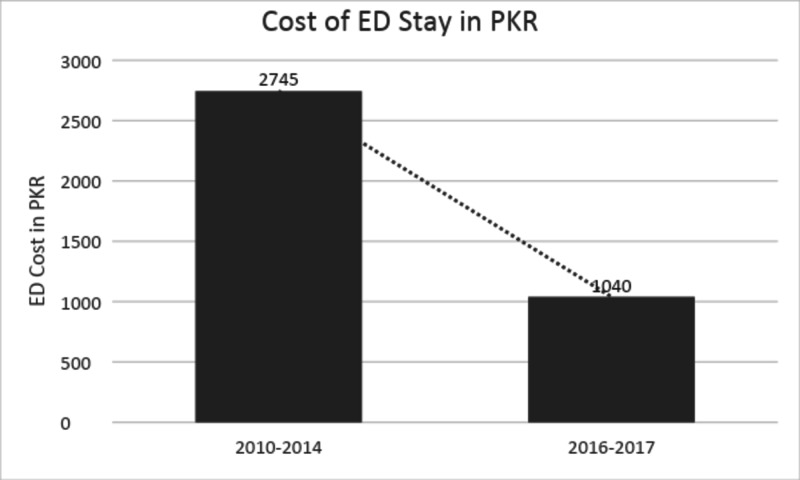
Trend in cost of ED stay in PKR ED: emergency department, PKR: Pakistani rupee.

The average ED length of stay of cardiac patients was decreased from 6.1 hours to 2.3 hours (Figure 3) and the cost of care for cardiac patients was reduced to 1040 PKR ($7) per hour from 2745 PKR ($18) per hour of ED stay as compared to year 2010-2014 institutional audit data results as shown in (Figure 4).

**Figure 5:**
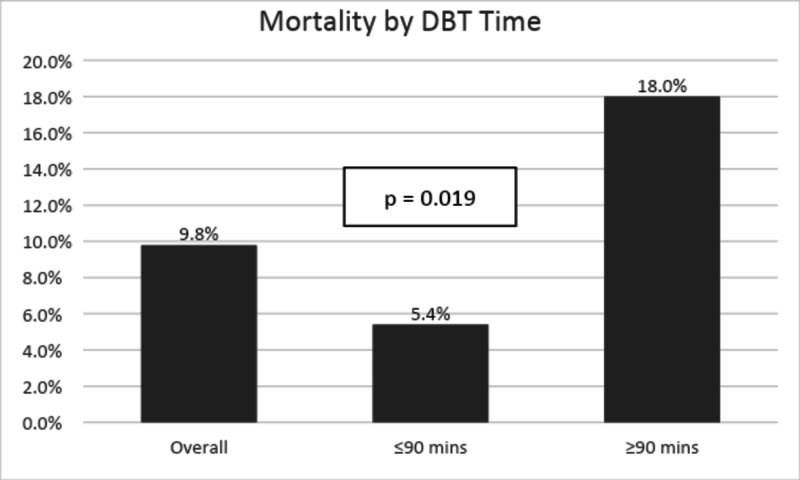
Trend in mortality by a change in DBT time DBT: door to balloon time.

A significant difference in hospital mortality of cardiac patients from 5.4% to 18% (p-valve =0.019) was observed between less than and more than 90 min of DBT, respectively (Figure 5).

**Figure 6:**
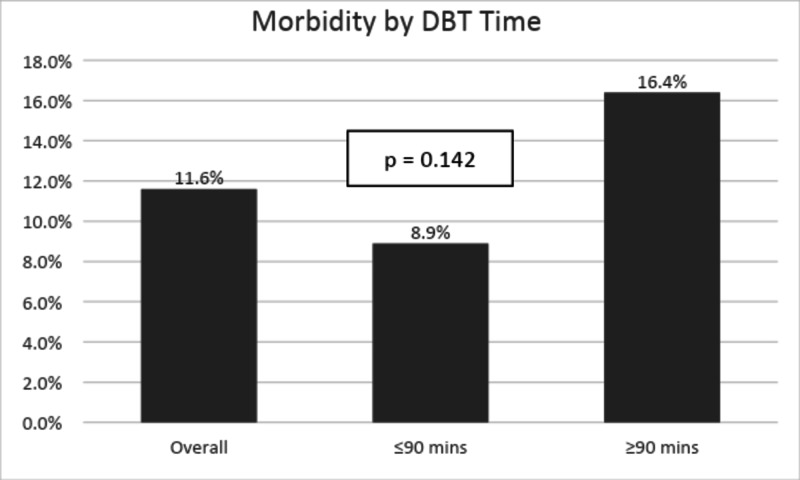
Trend in morbidity by a change in DBT time DBT: door to balloon time.

Overall morbidity is defined as a new onset of complications experienced during the hospital stay and includes major and minor morbidity [11] that was 11.6%; however, no significant difference was calculated between the groups (p-value = 0.142; Figure 6).

**Figure 7:**
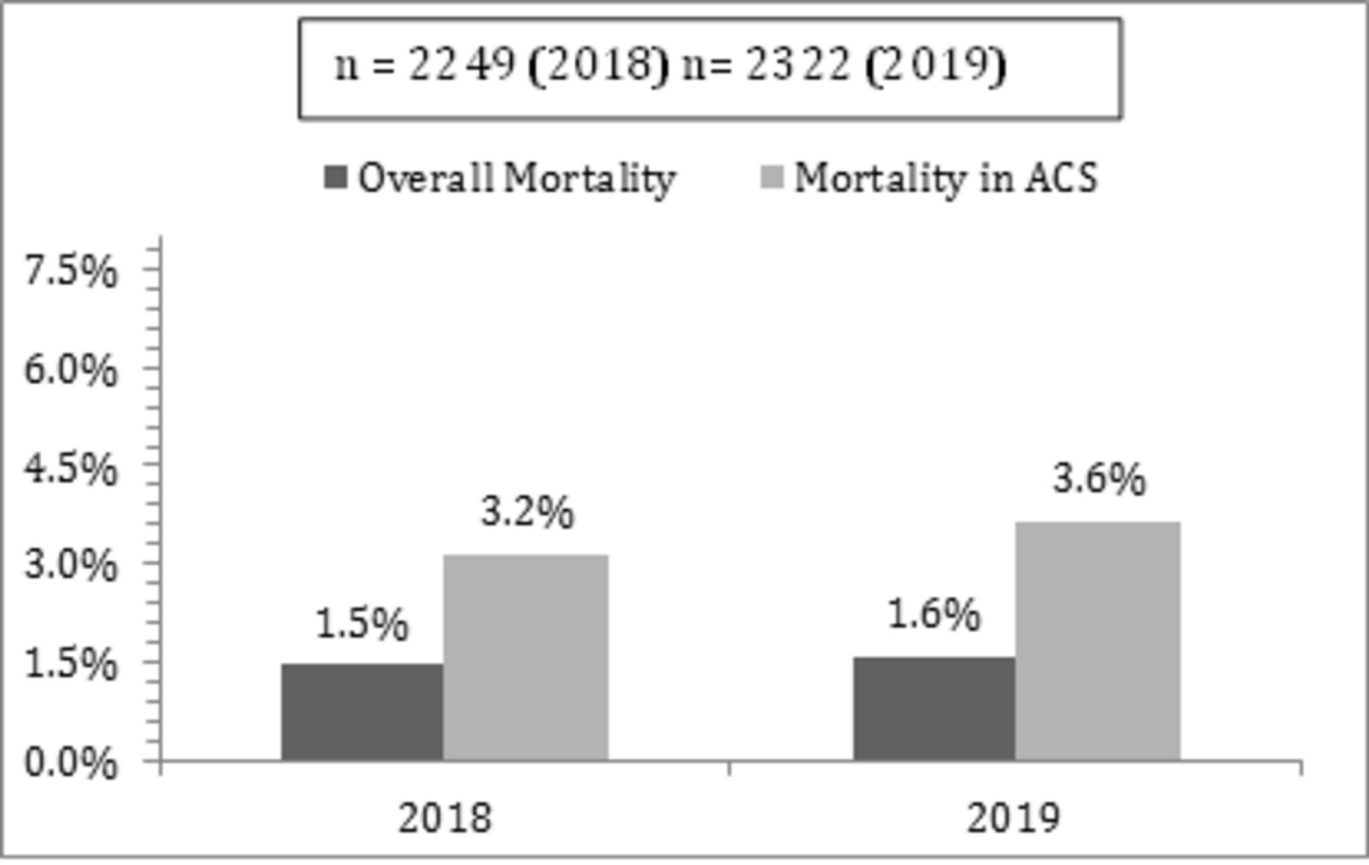
Overall and ACS mortality in 2018 and 2019 showing improvement as compared to 5.6% in 2017 ACS: acute coronary syndrome.

**Figure 8:**
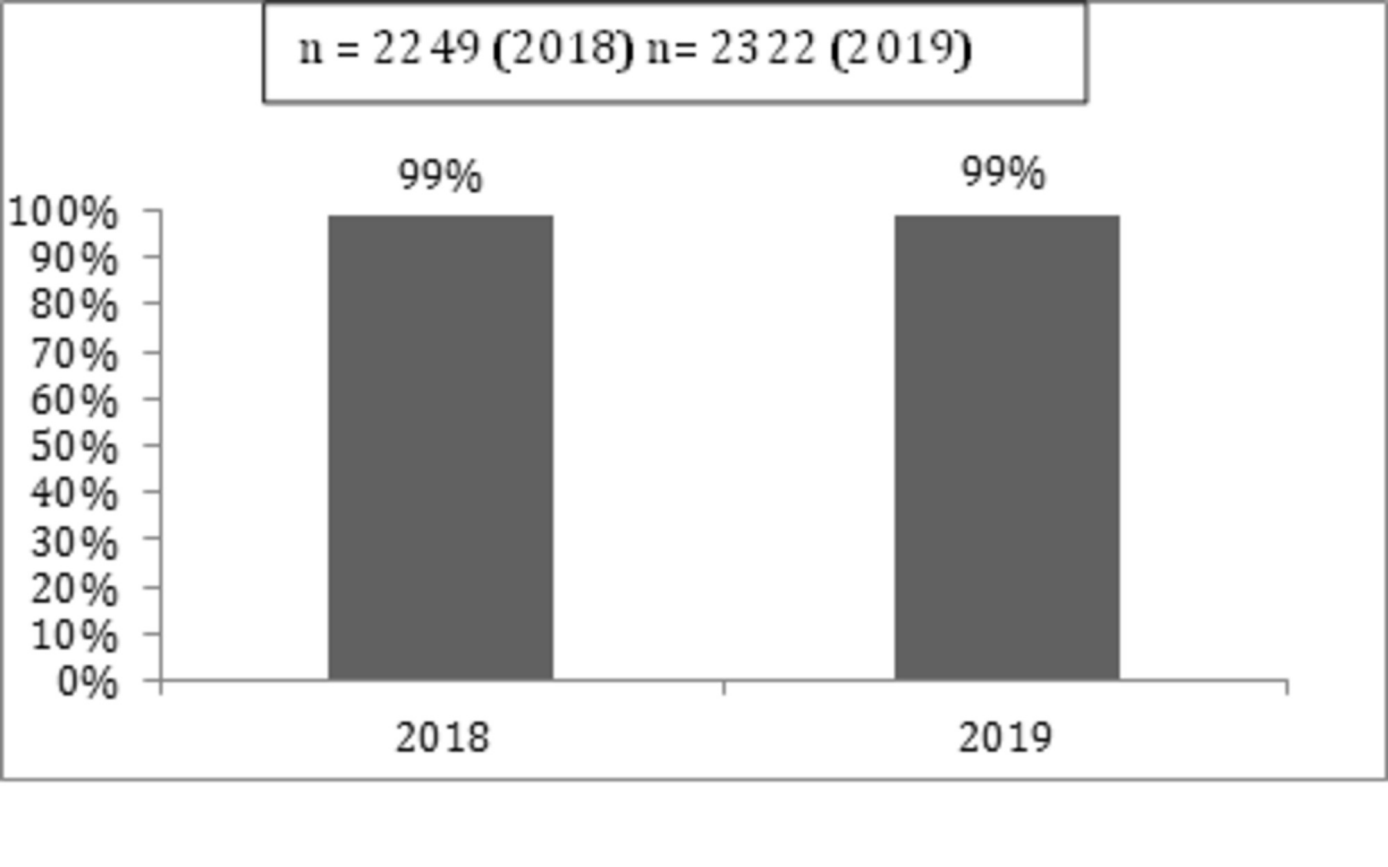
ECG within 10 minutes of arrival in CAR-ERU 2018 versus 2019 ECG: electrocardiogram, CAR-ERU: cardiac emergency unit.

**Figure 9:**
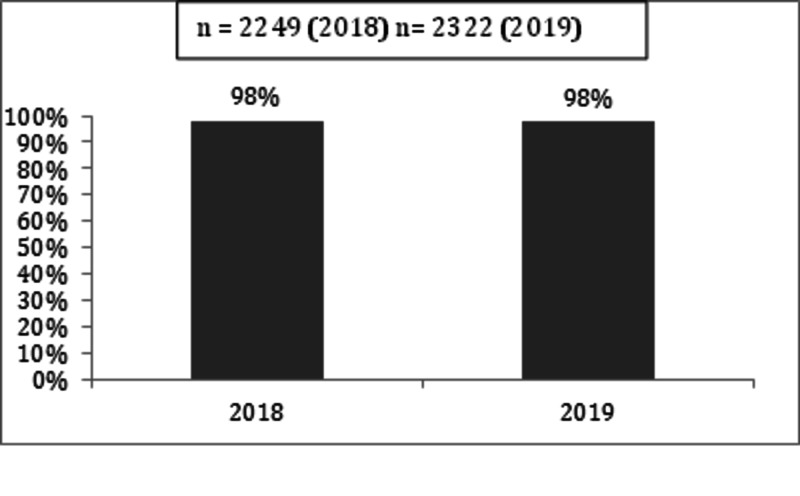
Aspirin within 60 minutes of arrival in CAR-ERU 2018 versus 2019 CAR-ERU: cardiac emergency unit.

**Figure 10:**
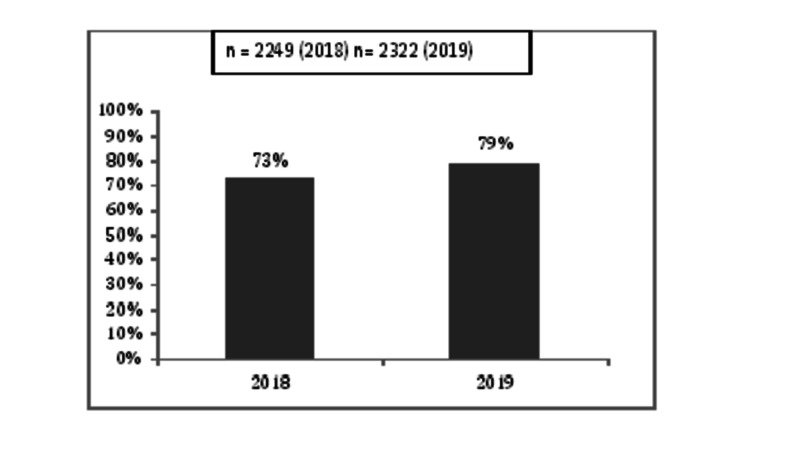
Door to balloon time <90 minutes 2018 versus 2019

To measure the sustainability of outcomes we continued to collect and analyse major KPIs over the year 2018 and 2019. The major KPIs measured and sustained over the period of 2018-2019 are shown in Figures 7-10.

## Discussion

In a recent qualitative study of third world countries like Pakistan, there are many obstacles in the way of providing good quality care to the patients; cost being one of them which not only hinders the delivery of care by healthcare professionals but also the adherence of the patients to their medications [10]. Hence, the quality projects like the establishment of a separate cardiac emergency unit within MED for early identification and management of patients with AMI, not only saves lives but also reduces financial expense and length of stay in ED.

 A study done at the Department of Medicine, University of California shows that in spite of providing the latest treatment options, the morbidity and mortality are high in missed myocardial infarction cases, and therefore, they have emphasized a standardized approach to the management of such cases [2]. The substantial decline in mortality cost borne by the patient and length of emergency stay in our data has supported these findings.

The European standard criteria for setting up a separate CAR-ERU as an integral part of MED are already set [11]. The criteria stated that patients with chest pain suspected of AMI should be directly triaged by the cardiac team in CAR-ERU without delay. The structure of our cardiac emergency unit meets with the standard criteria set for the chest pain unit. A dedicated and trained healthcare provider works in CAR-ERU with a strong collaboration among each other. The training for the staff is held every year along with monthly refresher courses.

Pre-hospital care is a neglected area in developing countries; hence, there could be a myriad of impacts on the patient outcome by quality improvement initiatives. A separate cardiac emergency unit can be linked to emergency medical services (EMS), facilitating pre-hospital identification of AMIs which can lead to timely revascularization and myocardial salvage [12].

Although our data have shown promising results, the study has certain limitations. The key performance indicators and outcomes are not stratified according to patient demographic factors, socioeconomic class, type and characteristic of myocardial infarction. Factors responsible for delayed revascularization in AMC, other than the delayed diagnosis in an emergency, such as lack of patient autonomy, family taking a decision on behalf of the patient, literacy rate, gender inequity and many more need to be identified and worked upon. A follow-up for five and ten years would better support the sustainability of parameters.

## Conclusions

The introduction of a dedicated CAR-ERU led to streamline the process of diagnosis and treatment of AMI patients, providing them the best quality of care and decreasing their morbidity, mortality, length of ED stay and financial burden. Not only was this project successful in establishing KPIs but it also showed better results in outcome matrices. The future implications of this quality project seem attainable and, if this is sustained can have an important effect on the patient’s quality of life.
